# Angiofibroma Localized in the Sphenoid Sinus

**DOI:** 10.1155/2017/4287293

**Published:** 2017-11-22

**Authors:** Alper Yenigun, Fadlullah Aksoy, Omer Vural, Orhan Ozturan

**Affiliations:** Department of Otorhinolaryngology, Faculty of Medicine, Bezmialem Vakif University, Fatih, Istanbul, Turkey

## Abstract

Juvenile nasopharyngeal angiofibroma is the most common benign tumor of the nasopharynx with complaints of unilateral nasal obstruction and recurrent nosebleeds in the young male population. Despite being a benign tumor, it can be aggressively destructive in surrounding tissues and bones by acting locally. The gold standard treatment method is the surgical excision of the tumor. This case report is a case of angiofibroma, a 32-year-old asymptomatic male patient with no evidence of clinical signs and endoscopic examination, which is recognized as a localized vascular mass lesion in the right sphenoid sinus on the cranial MR imaging. We prepared this case report that may represent an angiofibroma localized only within the sphenoid sinus which is very rare in the literature.

## 1. Introduction

Juvenile nasopharyngeal angiofibroma (JNA) usually destroys adolescent male patients aged 14–25 years and accounts for 0.5% of head and neck tumor [1]. JNA is histologically benign, noncapsular, and vascular-originated and develops in the nasopharynx, which tends to spread to surrounding tissues in all directions [2]. Usually, JNA is located on the superior edge of the sphenopalatine foramen; nonetheless, it can be seen at any localization in the nasopharynx and nasal cavity [3]. It is important to determine the roots of the JNA's origin in order to predict the direction of the growth [1]. In this case, a 32-year-old male patient with an angiofibroma-localized vascular mass lesion in the right sphenoid sinus on the cranial MR imaging without any clinical symptom and examination finding was presented.

## 2. Case Report

**Figure 1:**
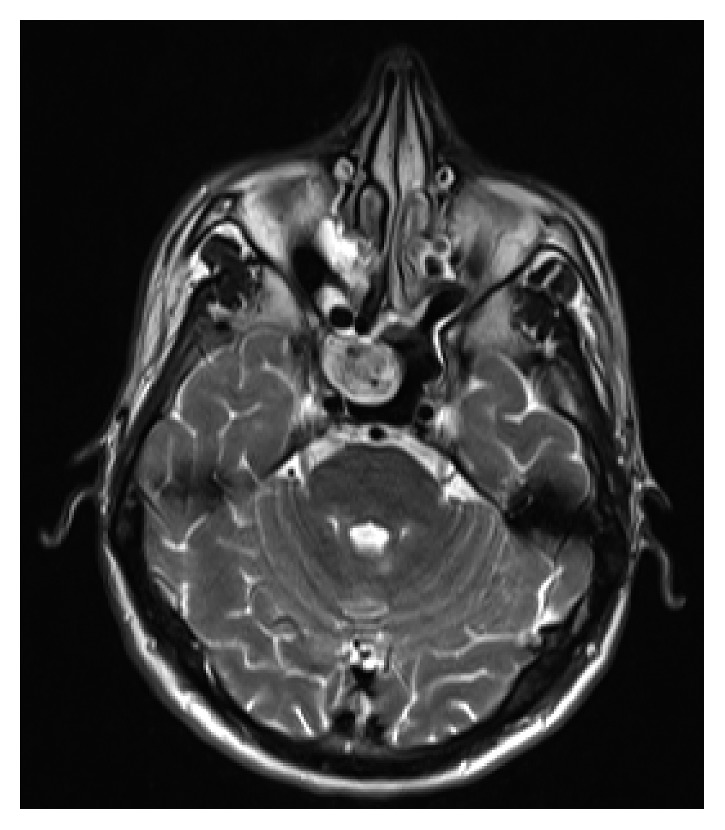
Magnetic resonance image (T-2) of angiofibroma localized in the sphenoid sinus.

**Figure 2:**
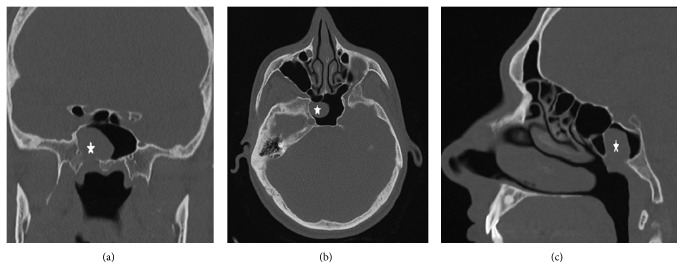
Computed tomography image of angiofibroma localized in the sphenoid sinus ((a) coronal, (b) axial, and (c) sagittal).

**Figure 3:**
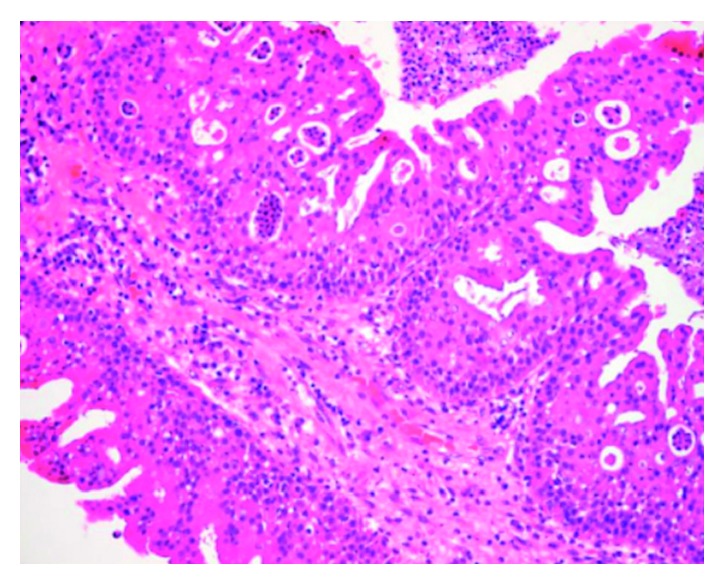
Pathological section view of angiofibroma.

A 32-year-old asymptomatic male patient who was followed up for multiple sclerosis in the neurology clinic was referred to ENT clinic. The cranial MR imaging revealed an intensely vascular lesion which contained hypointense areas with an expansile growth pattern within the right sphenoid sinus (Figure 1). Nasal endoscopic examination of the patient did not reveal any pathology, and contrast-enhanced paranasal sinus (PNS) CT scan was performed to evaluate the bony structure. The PNS CT scan revealed a soft tissue mass about 2 × 2 cm in dimensions in the lateral part of the right sphenoid sinus, extending to the pterygopalatine fossa showing intense contrast enhancement with bone destruction (Figure 2). The clinical examination and radiologic imaging suggested the diagnosis of JNA, and the patient was referred to the interventional radiology clinic. Under local anesthesia, the right internal maxillary artery (IMA) providing blood supply to the mass was embolized with PVA particles of 500 microns in size in the interventional radiology clinic. The mass was completely removed by the endonasal endoscopic procedure under general anesthesia twenty-four hours after this procedure. The patient's vital signs were stable, and there was no bleeding in the postoperative period. The specimen's pathological examination was reported to be compatible with angiofibroma (Figure 3). During the six months of follow-up of the patient, no complication and recurrence have occurred.

## 3. Discussion

JNA is a histologically benign, nonencapsulated, locally aggressive, and vascular-originated neoplasm and a rare tumor in adolescents [3, 4]. Although the pathogenesis of JNA is not fully understood, the fact that the disease is seen in adolescent males suggests that it may be caused by a problem in the hypophysial androgen-estrogen cycle [5]. JNA can lead to life-threatening complications such as fatal epistaxis, intracranial extension, and intraoperative hemorrhage [2]. In this case presentation, the patient was asymptomatic, and the endoscopic nasal examination of the patient was normal. There was a mass located in the right sphenoid sinus which was detected only by imaging methods taken for other reasons. Because the mass was localized within the right sphenoid sinus, there was no nosebleed history in the patient's story. For this reason, it was thought that the diagnosis could not be made in the adolescent age.

Despite the use of sclerosing agents, cryotherapy, hormonal therapy, and preoperative embolization in JNA treatment, the most effective treatment methods are nonsurgical techniques combined with radiotherapy and preoperative angiography. The gold standard method is the surgical excision of tumor [4]. JNA can cause a patient's life because it is vascular-originated and can lead to serious bleeding during surgical intervention [6]. Because of this, the most widely accepted method for reducing the risk of preoperative hemorrhage is the preoperative embolization of the tumor [6]. There is no consensus in the literature about the surgery time after embolization, but it is widely accepted that it should not exceed 48 hours [7]. An embolization performed 24–72 hours before the surgery has been shown to reduce blood loss during the surgery [8]. Embolization was performed 24 hours before the surgery in our case. Angiofibromas of the sphenoid sinus are extremely rare. These kinds of tumors are exciting because of the proximity of the internal carotid artery and optic nerve. Aksoy et al. showed that accessory sphenoidal septa can originate from the internal carotid artery or the optic nerve [9]. Therefore, the presence of an accessory sphenoidal septum increases the risk of surgical complications of angiofibroma in the sphenoid sinus. Internal carotid artery injury and loss of vision due to optic nerve damage may develop during the surgical intervention.

The nature of our case which is very rare in the literature, the JNA is completely localized within the sphenoid sinus. Therefore, it is diagnosed incidentally by imaging methods taken for other reasons as mass of the sphenoid sinus without any clinical symptoms and examination findings.
